# Recent Update Targeting Autophagy-Apoptosis Crosstalk Using Bioactive Natural Products for Ovarian Cancer Treatment

**DOI:** 10.3390/biomedicines14010212

**Published:** 2026-01-19

**Authors:** Abdel Halim Harrath, Maroua Jalouli, Mohammed Al-Zharani, Md Ataur Rahman

**Affiliations:** 1 Department of Zoology, College of Science, King Saud University, Riyadh 11451, Saudi Arabia; hharrath@ksu.edu.sa; 2 Department of Biology, College of Science, Imam Mohammad Ibn Saud Islamic University (IMSIU), Riyadh 11623, Saudi Arabia; mejalouli@imamu.edu.sa (M.J.); mmylzahrani@imamu.edu.sa (M.A.-Z.); 3 Department of Oncology, Karmanos Cancer Institute, Wayne State University, Detroit, MI 48201, USA

**Keywords:** autophagy, apoptosis, ovarian cancer, natural products, PI3K/AKT/mTOR signaling, chemotherapy

## Abstract

Ovarian cancer remains a top mortality contributor within gynecological cancers because patients receive diagnoses late in the disease course and conventional treatment resistance along with high recurrence rates cause poor outcomes. Aberrant regulation of autophagy and apoptosis has a critical role in the development, progression, chemoresistance, and immune escape from ovarian cancer. Recent evidence has demonstrated a complicated and dynamic crosstalk between autophagy and apoptosis, during which autophagy can act as a cytoprotective or cell death-promoting process depending on tumor stage and therapeutic context. In parallel, apoptosis functions as a tightly regulated form of programmed cell death that is essential for eliminating damaged or malignant cells and serves as a major tumor-suppressive mechanism in ovarian cancer. The PI3K/AKT/mTOR signaling pathway is the most active and clinically relevant pathway in the management of ovarian cancer as a master regulator of both autophagy and apoptosis, suppressing apoptotic cell death while promoting cytoprotective autophagy under chemotherapeutic stress. Bioactive natural products derived from plants, marine sources, and dietary intake have emerged as potential modulators of the autophagy-apoptosis crosstalk. Curcumin, resveratrol, quercetin, berberine, and epigallocatechin gallate are known to have the ability to restore apoptotic signaling, block pro-survival autophagy, and sensitize ovarian cancer cells to chemotherapy through the regulation of key pathways including PI3K/AKT/mTOR, AMPK, MAPK, p53, and Bcl-2 family proteins. In this review, we provide an updated understanding of the molecular mechanisms through which bioactive natural products modulate autophagy–apoptosis crosstalk in ovarian cancer. We also highlight the translational challenges, therapeutic potential, and future directions for the integration of natural product-based strategies in precision medicine for ovarian cancer.

## 1. Introduction

Ovarian cancer (OC) is one of the most aggressive and lethal gynecological malignancies, with an unacceptably high mortality rate worldwide [1]. Absence of early clinical symptoms and lack of reliable diagnostic biomarkers often lead to delayed diagnosis with established peritoneal dissemination and chemoresistance [2]. Despite recent improvements in surgical techniques and the use of platinum- and taxane-based chemotherapy, the five-year survival rate for women with advanced-stage OC is still not satisfactory. Tumor heterogeneity, stress-induced adaptive responses, and resistance to apoptosis have been attributed to treatment failure.

Autophagy and apoptosis are two highly regulated and evolutionarily conserved cell death processes determining cell fate during homeostasis and pathologic conditions [3]. Apoptosis is a key tumor suppressive process that protects the organism from genetically unstable or damaged cells, while autophagy is a dual-edged sword that promotes cell survival by maintaining cellular homeostasis through lysosomal degradation of damaged organelles and proteins [4]. Alterations in these programs in OC lead to tumorigenesis, metabolic reprogramming, survival in hypoxic and nutrient-deprived microenvironments, and therapeutic resistance [5]. Accumulating evidence now points toward a protective role of autophagy in blocking apoptosis in response to therapeutic insults, thus promoting cancer cell survival and disease relapse.

Bioactive natural chemicals have shown efficacy in modulating the interaction between autophagy and apoptosis in ovarian cancer. These encompass polyphenols and flavonoids, including curcumin, resveratrol, quercetin, luteolin, and epigallocatechin gallate, which are extensively documented to modulate the PI3K/AKT/mTOR, AMPK, MAPK, and p53 signaling pathways [6]. Alkaloids, such as berberine and analogous isoquinoline derivatives, demonstrate significant influence on mitochondrial apoptosis and stress-induced autophagy [7]. Terpenoids, including tanshinones and betulinic acid derivatives, affect mitochondrial integrity, oxidative stress, and survival signaling pathways [8]. Moreover, isothiocyanates such as sulforaphane and other sulfur-containing substances affect redox equilibrium, histone modification, and apoptotic susceptibility [9]. These structurally varied natural compounds collectively offer multi-target regulation capabilities over autophagy and apoptosis, providing mechanistic and translational insights for ovarian cancer treatment.

Interplay between autophagy and apoptosis is complex and tightly regulated by shared signaling mediators, such as Bcl-2 family proteins, Beclin-1, p53, caspases, and important upstream signaling pathways including PI3K/AKT/mTOR, AMPK, MAPK, and NF-κB [10]. Therefore, targeting the crosstalk between these signaling networks has emerged as a plausible strategy to sensitize OC cells to therapy. In this regard, bioactive natural products derived from plants, dietary sources, and marine organisms have attracted special interest due to their multimodal actions, favorable safety profiles, and ability to modulate complex signaling events [11]. Curcumin, resveratrol, quercetin, berberine, and epigallocatechin gallate (EGCG) are some of the bioactive natural compounds with potent anti-OC activity, which can re-establish apoptotic signaling and modulate autophagy [12]. Herein, we highlight recent advances in modulating the crosstalk between autophagy and apoptosis by bioactive natural compounds as a novel therapeutic strategy for OC management.

## 2. Molecular Crosstalk Between Autophagy and Apoptosis in Ovarian Cancer

**Figure 1:**
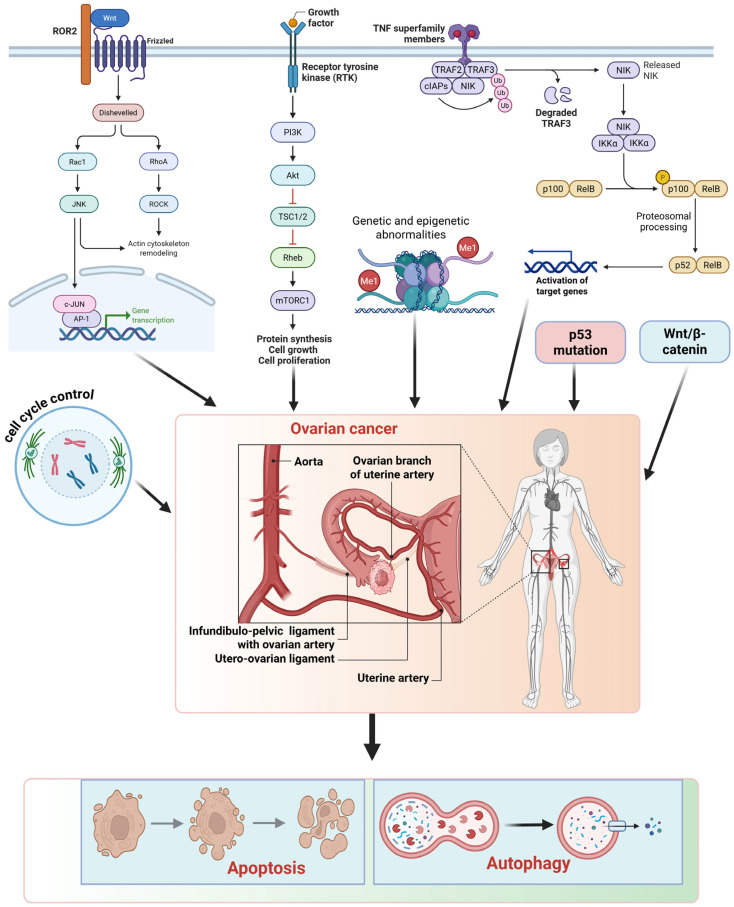
Molecular mechanisms of ovarian cancer interplay between apoptosis and autophagy. Inappropriate activation of oncogenic pathways, such as Wnt signaling through ROR2–Frizzled receptors, PI3K/AKT/mTOR signaling downstream of receptor tyrosine kinases, and non-canonical NF-κB signaling mediated by TNF superfamily members, enhances cell proliferation, survival, and cytoskeletal reorganization. Genetic and epigenetic anomalies, along with prevalent p53 mutations and dysregulated Wnt/β-catenin signaling, exacerbate unregulated cell cycle progression and tumor proliferation. These carcinogenic signals combine at the ovarian tissue level, facilitating tumorigenesis and angiogenesis. At the cellular level, ovarian cancer cells circumvent programmed cell death by inhibiting apoptosis through an altered balance of Bcl-2 family proteins and impaired caspase activation, while concurrently enhancing autophagy as an adaptive survival strategy in response to metabolic and therapeutic stress. The synchronized regulation and functional interaction between apoptosis and autophagy allow ovarian cancer cells to sustain homeostasis, withstand chemotherapy, and facilitate disease advancement.

Ovarian cancer development and progression are driven by complex molecular alterations that disrupt normal cellular functions, such as cell cycle regulation, DNA repair, metabolism, and apoptosis [13]. Genetic and epigenetic alterations, including mutations in TP53 and BRCA1/2 genes, and the dysregulation of homologous recombination repair pathways, contribute to genomic instability and tumor formation [14]. The activation of oncogenic signaling pathways, such as PI3K/AKT/mTOR, MAPK, Wnt/β-catenin, and NF-κB, promotes uncontrolled cell proliferation, metabolic reprogramming, angiogenesis, and invasion [15]. Ovarian cancer cells often evade apoptosis by upregulating anti-apoptotic Bcl-2 family proteins, inactivating caspases, and disrupting p53-mediated cell death signaling [16]. Autophagy is often upregulated in ovarian cancer as a protective mechanism, enabling tumor cells to survive under conditions of hypoxia, nutrient deprivation, and chemotherapy-induced stress [17]. The crosstalk between autophagy and apoptosis, mediated by shared regulators such as Beclin-1, Bcl-2, and mTOR, contributes to tumor cell survival and resistance to therapy (Figure 1). These interconnected molecular processes contribute to the aggressive nature of ovarian cancer and represent important targets for therapeutic intervention.

### 2.1. Autophagy in Ovarian Cancer

**Figure 2:**
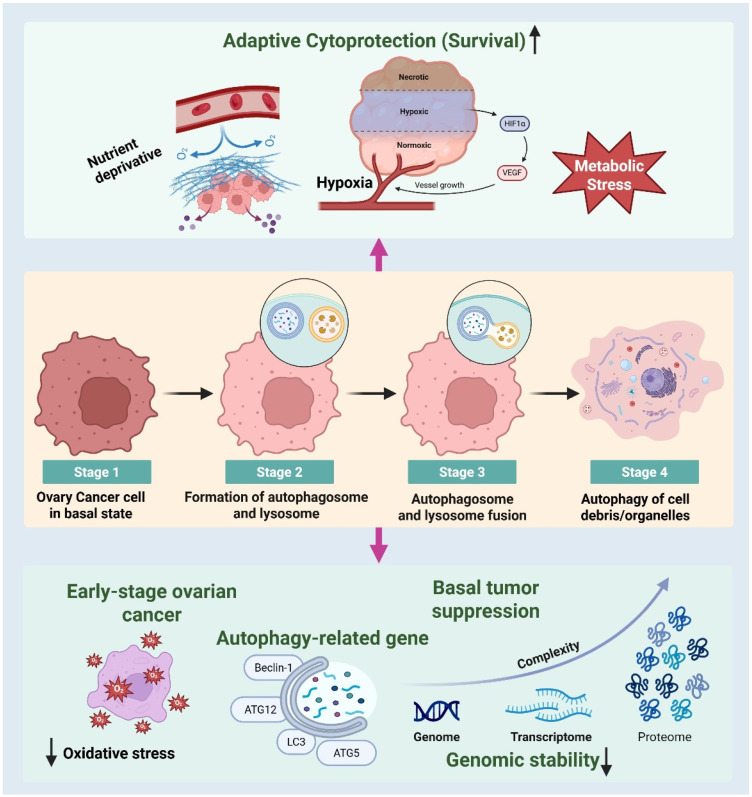
Autophagy in ovarian cancer: dual roles in tumor suppression and adaptive cytoprotection. The context-specific roles of autophagy in the initiation and progression of ovarian cancer. In established ovarian cancers, detrimental microenvironmental factors such as nutritional deficiency, hypoxia, and metabolic stress trigger autophagy as an adaptive cytoprotective process that enhances cancer cell survival. Hypoxia-inducible factor-1α (HIF-1α) signaling promotes angiogenesis and metabolic adaptability, hence supporting tumor survival under stress conditions. Conversely, in early-stage ovarian cancer, basal autophagy has a tumor-suppressive role by mitigating oxidative stress and preserving cellular homeostasis. Essential autophagy-related genes, including Beclin-1, ATG12, ATG5, and LC3, play a pivotal role in maintaining genomic stability through the regulation of genome integrity, transcriptome equilibrium, and proteomic quality assurance. The dynamic equilibrium between autophagy-mediated tumor suppression in early-stage disease and autophagy-facilitated survival in advanced ovarian cancer underscores autophagy as a vital and stage-specific therapeutic target.

Autophagy is a highly conserved lysosome-dependent catabolic process that is critical for maintaining cellular homeostasis through the recycling of damaged organelles and misfolded proteins [18]. In ovarian cancer, autophagy has context-dependent roles in tumorigenesis and cancer development, functioning as both a tumor suppressor and a tumor promoter [19]. During the early stages of carcinogenesis, basal autophagy supports genomic stability by reducing oxidative stress and preventing mitochondrial dysfunction [20]. In established ovarian cancers, autophagy is frequently upregulated and promotes cancer survival under adverse conditions such as hypoxia, nutrient deprivation, and metabolic stress [19]. Increased autophagic flux enables ovarian cancer cells to adapt to the tumor microenvironment and resist cytotoxicity induced by therapy (Figure 2). Critical autophagy-related genes, including Beclin-1, LC3, ATG5, and ATG7, are often dysregulated in ovarian cancer and are associated with disease progression and poor prognosis [21]. The activation of autophagy is primarily controlled by signaling pathways such as PI3K/AKT/mTOR, AMPK, MAPK, and HIF-1α, which integrate environmental cues and cellular stress signals [22]. Chemotherapeutic drugs, particularly platinum-based drugs, can induce protective autophagy, leading to decreased apoptosis and drug resistance [23]. Therefore, targeting autophagy has emerged as a potential strategy to enhance therapeutic efficacy in ovarian cancer. Understanding the molecular regulation of autophagy is essential to exploit its dual role and identify optimal windows for therapeutic intervention.

### 2.2. Apoptosis in Ovarian Cancer

**Figure 3:**
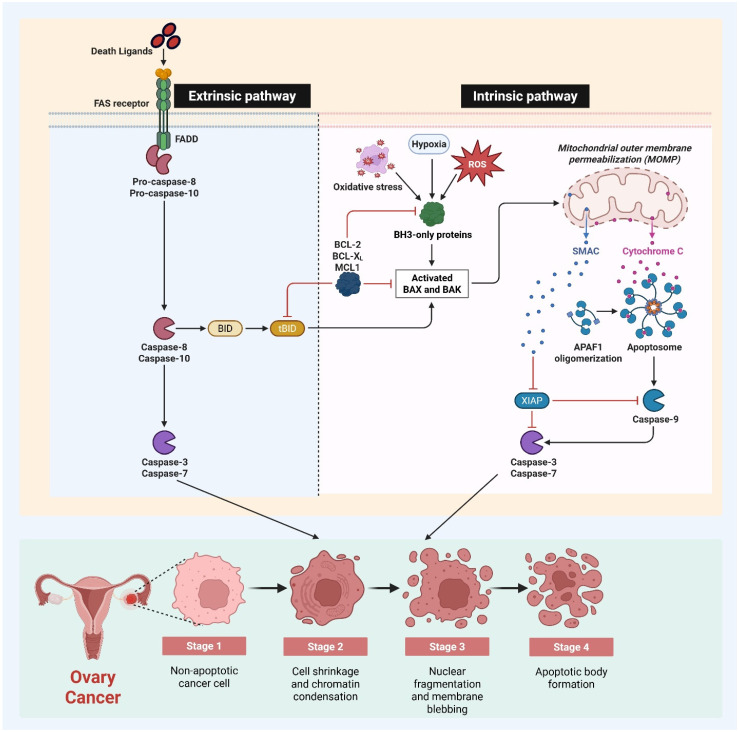
Apoptosis in ovarian cancer: dysregulation of intrinsic and extrinsic apoptotic pathways. The extrinsic route commences when death ligands attach to the Fas receptor, resulting in the recruitment of FADD and the activation of pro-caspase-8 and pro-caspase-10. Activated caspase-8 directly initiates executioner caspases-3 and -7 and cleaves BID to form truncated BID (tBID), establishing a molecular connection between extrinsic and intrinsic apoptosis. The intrinsic mitochondrial route is initiated by cellular stresses, including hypoxia, oxidative stress, and reactive oxygen species, which activate BH3-only proteins. These signals facilitate the activation of BAX and BAK, leading to the permeabilization of the mitochondrial outer membrane and the release of cytochrome c and SMAC. Cytochrome c promotes the oligomerization of APAF1 and the development of the apoptosome, resulting in the activation of caspase-9 and subsequently the executioner caspases-3 and -7. Anti-apoptotic proteins, such as BCL-2, BCL-XL, MCL1, and XIAP, inhibit caspase activity and apoptotic signaling, thus fostering apoptosis resistance in ovarian cancer cells. The lower panel illustrates the morphological steps of apoptosis, advancing from non-apoptotic ovarian cancer cells to cell shrinkage, chromatin condensation, nuclear fragmentation, membrane blebbing, and the creation of apoptotic bodies. This together emphasizes the foundation of disrupted apoptosis in ovarian cancer and identifies critical areas for therapeutic intervention.

Apoptosis is a highly regulated form of programmed cell death that plays a crucial role in eliminating damaged or abnormal cells, and acts as an important tumor-suppressive mechanism [24]. Dysregulation of apoptotic signaling pathways is a hallmark of ovarian cancer that contributes to uncontrolled cell proliferation, genomic instability, and drug resistance [25]. Both intrinsic and extrinsic apoptotic pathways are often disrupted in ovarian cancers. The intrinsic mitochondrial pathway is commonly affected by alterations in the expression of Bcl-2 family proteins, leading to the overexpression of anti-apoptotic proteins such as Bcl-2 and Bcl-xL, and the downregulation of pro-apoptotic proteins like Bax and Bak [26]. In addition, mutations or inactivation of tumor suppressor genes, particularly p53, further impair apoptotic responses and contribute to tumorigenesis. Activation of caspases, a key step in the apoptotic process, is often reduced in ovarian cancer, leading to decreased sensitivity to chemotherapy-induced cell death [27]. The extrinsic apoptotic pathway, mediated by death receptors such as Fas and Tumor Necrosis Factor-related Apoptosis-inducing Ligand (TRAIL) receptors, is also disrupted due to receptor downregulation or defects in downstream signaling [28]. Resistance to apoptosis is a significant challenge in the treatment of ovarian cancer, as many traditional chemotherapeutic agents primarily rely on the induction of apoptosis for their cytotoxic effects (Figure 3). Targeting and restoring apoptotic signaling has therefore become an important therapeutic strategy in the management of ovarian cancer.

### 2.3. Autophagy-Apoptosis Interplay in Ovarian Cancer Progression

The crosstalk between autophagy and apoptosis plays an important role in ovarian cancer cell fate decisions, influencing tumorigenesis, metastasis, and response to therapy. Autophagy and apoptosis are interconnected through several molecular regulators, which can shift the balance between cell survival and cell death [29]. For instance, Beclin-1, a key autophagy initiator, directly interacts with the anti-apoptotic proteins Bcl-2 and Bcl-xL, linking autophagy to the suppression of apoptosis [30]. Disrupting this interaction can simultaneously induce both autophagy and apoptosis, highlighting the complex interplay between these two processes. Regulation of the autophagy-apoptosis axis in ovarian cancer cells is also orchestrated by key signaling pathways such as PI3K/AKT/mTOR, AMPK, MAPK, and p53 [31]. Under therapeutic stress, autophagy is often regulated as a pro-survival mechanism to delay or prevent apoptosis. However, prolonged or excessive autophagy may lead to autophagy-dependent cell death and promote apoptotic signaling [32]. This plasticity allows ovarian cancer cells to modulate their response to chemotherapy, contributing to treatment resistance and disease relapse. Targeting the autophagy-apoptosis crosstalk represents a promising strategy to overcome treatment resistance by inhibiting cytoprotective autophagy and restoring apoptotic sensitivity [33]. A better understanding of this regulatory network is essential for developing effective combination therapies for ovarian cancer.

The synergistic interaction between autophagy and apoptosis is crucial in inhibiting growth and metastasis in ovarian cancer. Under normal settings, regulated autophagy and apoptosis preserve cellular homeostasis and remove damaged or altered cells. In ovarian carcinogenesis, the deregulation of this cascade allows cancer cells to circumvent programmed cell death and maintain survival amid metabolic and therapeutic stress [34]. The therapeutic reinstatement of apoptosis, together with the inhibition of cytoprotective autophagy, reinstates cell death signaling and curtails malignant transformation [35]. Prolonged or excessive autophagy can shift from a survival mechanism to autophagy-induced cell death, effectively eradicating apoptosis-resistant cancer cells. At the metastatic stage, the manipulation of the autophagy-apoptosis axis disrupts epithelial–mesenchymal transition, anoikis resistance, and adaptation to the metastatic niche [36]. Crosstalk facilitated by common regulators, including Bcl-2, Beclin-1, p53, and the PI3K/AKT/mTOR signaling pathway, impedes cytoskeletal reorganization, invasion, and survival in circulation [37]. Targeting the autophagy-apoptosis cascade substantially inhibits ovarian cancer initiation, development, and metastatic spread by concurrently disrupting survival pathways and enhancing programmed cell death.

### 2.4. Mitochondria-Mediated Apoptosis-Autophagy in Ovarian Cancer Microenvironment

Mitochondria-mediated apoptosis and autophagy are intricately linked mechanisms that significantly influence the ovarian cancer microenvironment and determine tumor cell fate. Mitochondria serve as pivotal centers for intrinsic apoptotic signaling by facilitating mitochondrial outer membrane permeabilization, resulting in cytochrome c release, apoptosome assembly, and caspase activation [38]. Dysregulation of Bcl-2 family proteins in ovarian cancer cells disrupts this mechanism and fosters resistance to apoptosis [39]. Mitophagy, a selective autophagic process that removes damaged mitochondria, contributes to this equilibrium by maintaining mitochondrial integrity and curtailing excessive ROS generation. In the ovarian tumor microenvironment, hypoxia and metabolic stress induce mitophagy via HIF-1α- and AMPK-dependent pathways, allowing cancer cells to endure adverse conditions [40]. Excessive or impaired mitophagy can lead to mitochondrial malfunction and activate apoptotic signals [41]. Additionally, stromal and immunological cells in the microenvironment employ mitochondrial autophagy to regulate inflammation, angiogenesis, and redox equilibrium, hence indirectly influencing apoptotic sensitivity in ovarian cancer cells [42]. The mitochondria-centered apoptosis-autophagy axis constitutes a vital regulatory network that affects tumor development, immune evasion, and treatment response in ovarian cancer.

## 3. Bioactive Natural Products Targeting Autophagy-Apoptosis Interplay in Ovarian Cancer

### 3.1. Bioactive Natural Products Modulating Autophagy in Ovarian Cancer

Autophagy modifiers from natural sources with low toxicity are attractive candidates for cancer therapy. Autophagy is regulated by bioactive natural compounds in a highly context-dependent manner. It can be either inhibited (protective autophagy) or induced to a lethal level (excessive autophagic flux). Many bioactive natural compounds have been shown to inhibit pro-survival autophagy associated with the progression of ovarian cancer and chemoresistance [43]. Curcumin, a polyphenol derived from *Curcuma longa*, inhibits the PI3K/AKT/mTOR pathway, which leads to a decrease in autophagosome production and an increase in chemosensitivity [44]. Resveratrol also suppresses mTOR signaling and activates AMPK, which impairs metabolic adaptation and limits protective autophagy in ovarian cancer cells [45]. Flavonoids such as quercetin and kaempferol regulate autophagy by modulating the expression of Beclin-1, LC3, and p62 [46]. This results in changes in autophagic flux and decreased viability of tumor cells. Berberine, an isoquinoline alkaloid, induces autophagy-mediated cell death through mitochondrial dysfunction and AMPK activation, particularly in drug-resistant ovarian cancer models [47]. Epigallocatechin gallate, a major catechin found in green tea, disrupts lysosomal function and autophagosome maturation, increasing the sensitivity of ovarian cancer cells to platinum-based chemotherapy [48]. These compounds target key regulators of autophagy such as mTOR, AMPK, HIF-1α, and Beclin-1, and therefore can regulate tumor metabolism and stress responses. Bioactive natural compounds may represent a promising therapeutic strategy as an adjunct to current standard of care for ovarian cancer by modulating autophagic activity (Table 1).

**Table 1:** Bioactive natural products that modulate autophagy in ovarian cancer.

Bioactive Natural Product	Major Molecular Targets and Pathways	Experimental Model	Dose Range	Autophagy Effect	Key Autophagy Markers	Autophagy Flux Validation	Therapeutic Implication	Ref.
**Curcumin**	PI3K/AKT/mTOR inhibition, AMPK activation	A2780, SKOV3	5–40 µM	Induction (often cytotoxic)	↑LC3-II, ↑Beclin-1, ↓p62	CQ, 3-MA, LC3 puncta	Enhances chemosensitivity, multi-target agent	[49]
**Resveratrol**	ROS generation, AMPK activation, mTOR suppression	SKOV3, OVCAR3	10–100 µM	Induction	↑LC3-II, ↑Atg5	CQ, bafilomycin A1	Couples’ autophagy with apoptosis	[50]
**Quercetin**	PI3K/AKT inhibition, NF-κB suppression	A2780, SKOV3	10–50 µM	Modulation	↑LC3-II, ↓p62	LC3 puncta	Suppresses survival signaling	[51]
**EGCG**	PI3K/AKT inhibition, lysosomal stress	SKOV3, OVCAR3	20–80 µM	Modulation	↑LC3-II, LAMP1 changes	Partial flux assays	Chemosensitizing potential	[52]
**Berberine**	AMPK activation, mitochondrial stress	A2780, OVCAR3	5–40 µM	Induction (autophagic cell death)	↑LC3-II, ↑Beclin-1	CQ, Atg5 knockdown	Effective in resistant cells	[53]
**Genistein**	AKT inhibition, metabolic stress	A2780, ES-2	10–50 µM	Induction	↑LC3-II, ↑Beclin-1	3-MA	Targets metabolic adaptation	[54]
**Isoliquiritigenin**	ROS-mediated stress, caspase signaling	OVCAR5, ES-2	5–30 µM	Induction	↑LC3-II, ↓p62	LC3 puncta	Dual autophagy-apoptosis activation	[55]
**Baicalein**	ERK activation, Beclin-1 regulation	OVCAR3, CP70	10–40 µM	Induction	↑LC3-II, ↑Beclin-1	CQ	Effective in platinum resistance	[56]
**Baicalin**	NF-κB and survival pathway inhibition	OVCAR3, CP70	20–80 µM	Induction	↑LC3-II	Limited flux assays	Lower toxicity profile	[57]
**Paeonol**	AKT/mTOR blockade	A2780, SKOV3	50–200 µM	Induction (cytoprotective)	↑LC3-II, ↓p62	3-MA	Combination with autophagy inhibitors	[58]
**Ellagic acid**	AMPK activation, AKT inhibition	SKOV3	10–60 µM	Induction	↑LC3-II, ↑Beclin-1	LC3 puncta	Metabolic stress targeting	[59]
**Sulforaphane**	miRNA regulation, oxidative stress	A2780, IGROV1	2–20 µM	Context dependent	LC3-II changes	CQ, gene silencing	Reverses cisplatin resistance	[60]
**Tanshinone I**	PI3K/AKT/mTOR modulation	A2780, ID-8	1–10 µM	Induction	↑LC3-II, ↑Atg7	CQ	Autophagy-associated cell death	[61]
**Grifolin**	AKT/mTOR/S6K suppression	A2780, SKOV3	5–25 µM	Induction (cytotoxic)	↑LC3-II, ↓p62	Atg7 knockdown	Potent growth suppression	[62]
**Proanthocyanidins**	ROS and HIF-1α suppression	SKOV3, CP70	10–100 µg/mL	Modulation	LC3-II, p62 changes	Partial flux	Targets hypoxia-driven resistance	[63]

### 3.2. Bioactive Natural Products Inducing Apoptosis in Ovarian Cancer

Induction of apoptosis is another primary mechanism through which bioactive natural compounds exert anti-ovarian cancer activities. Many natural compounds effectively restore defective apoptotic signaling pathways that are often dysregulated in ovarian cancers. Curcumin promotes mitochondrial-dependent apoptosis by increasing the Bax/Bcl-2 ratio, inducing cytochrome c release, and activating caspase-9 and caspase-3 [64]. Resveratrol triggers intrinsic apoptosis through p53 activation and mitochondrial membrane depolarization, leading to cell cycle arrest and cancer cell death [65]. Flavonoids such as quercetin and luteolin induce apoptosis by repressing anti-apoptotic proteins, including Bcl-2 and survivin, and promoting caspase activation [66]. These compounds also inhibit NF-κB signaling, an important pathway implicated in apoptosis resistance in ovarian cancer. Berberine induces both intrinsic and extrinsic apoptotic pathways by activating death receptors and facilitating caspase-8 cleavage, as well as inducing mitochondrial dysfunction [67]. Epigallocatechin gallate promotes apoptosis through modulation of oxidative stress, inhibition of PI3K/AKT signaling, and activation of caspase cascades [68]. Other natural compounds, like genistein and sulforaphane, induce apoptosis by modulating estrogen receptor signaling, histone deacetylases, and redox homeostasis, contributing to the elimination of ovarian cancer cells [69]. Importantly, many of these compounds selectively induce apoptosis in cancer cells while sparing normal ovarian epithelial cells. Bioactive natural compounds can enhance the efficacy of conventional chemotherapy regimens and overcome therapy resistance in ovarian cancer by restoring apoptotic competence and circumventing survival signaling (Table 2).

**Table 2:** Bioactive natural products inducing apoptosis in ovarian cancer.

Bioactive Natural Product	Major Apoptotic Molecular Mechanisms	Experimental Model	Dose Range	Apoptotic Pathway	Key Apoptosis Markers	Therapeutic Implication	Ref.
**Curcumin**	Increases Bax/Bcl-2 ratio, mitochondrial depolarization, caspase activation	A2780, SKOV3, OVCAR3	5–40 µM	Intrinsic (mitochondrial)	↑Cleaved caspase-3/9, ↑PARP cleavage	Enhances platinum sensitivity	[70]
**Resveratrol**	p53 activation, ROS generation, cytochrome c release	SKOV3, CAOV3	10–100 µM	Intrinsic	↑Bax, ↓Bcl-2, ↑caspase-3	Targets p53-dependent death	[71]
**Quercetin**	NF-κB inhibition, survivin suppression	A2780, OVCAR3	10–50 µM	Intrinsic	↑Cleaved caspase-3, ↓survivin	Overcomes apoptosis resistance	[72]
**Berberine**	Mitochondrial dysfunction, death receptor activation	A2780, SKOV3	5–40 µM	Intrinsic and extrinsic	↑Caspase-3/8, ↑PARP	Effective in resistant cells	[73]
**EGCG**	AKT inhibition, oxidative stress induction	SKOV3, OVCAR3	20–80 µM	Intrinsic	↑Cleaved caspase-3, ↑Bax	Chemosensitizing agent	[74]
**Genistein**	Estrogen receptor modulation, caspase activation	A2780, ES-2	10–50 µM	Intrinsic	↑Caspase-3, ↓Bcl-2	Targets hormone-linked signaling	[75]
**Luteolin**	PI3K/AKT inhibition, ROS-mediated apoptosis	SKOV3, OVCAR3	10–40 µM	Intrinsic	↑Caspase-3, ↑PARP cleavage	Suppresses survival pathways	[76]
**Apigenin**	p53 upregulation, cell cycle arrest	A2780, CAOV3	10–50 µM	Intrinsic	↑Bax, ↑caspase-9	Low toxicity profile	[77]
**Baicalein**	Mitochondrial membrane disruption	OVCAR3, CP70	10–40 µM	Intrinsic	↑Cytochrome c, ↑caspase-3	Active in platinum resistance	[78]
**Baicalin**	NF-κB suppression, oxidative stress	OVCAR3, CP70	20–80 µM	Intrinsic	↑Cleaved caspase-3	Selective tumor toxicity	[79]
**Isoliquiritigenin**	ROS-dependent apoptosis, PARP cleavage	ES-2, OVCAR5	5–30 µM	Intrinsic	↑PARP, ↑caspase-3	Dual autophagy-apoptosis effect	[80]
**Sulforaphane**	HDAC inhibition, mitochondrial stress	A2780, IGROV1	2–20 µM	Intrinsic	↑Bax, ↑caspase-3	Sensitizes cisplatin-resistant cells	[81]
**Piperlongumine**	ROS accumulation, antioxidant system inhibition	SKOV3	2–10 µM	Intrinsic	↑Cleaved caspase-3	Targets redox vulnerability	[82]
**Tanshinone IIA**	PI3K/AKT suppression, mitochondrial damage	A2780, ID-8	1–10 µM	Intrinsic	↑Caspase-3/9, ↓Bcl-2	Anti-tumor and anti-metastatic	[83]
**Betulinic acid**	Direct mitochondrial permeabilization	SKOV3, OVCAR3	5–30 µM	Intrinsic	↑Cytochrome c, ↑caspase-3	Potent mitochondria-targeted agent	[84]

## 4. Recent Therapeutic Implications and Clinical Application of Bioactive Natural Products Targeting Autophagy-Apoptosis Interplay in Ovarian Cancer

Emerging evidence has highlighted the potential importance of targeting the autophagy-apoptosis crosstalk as a strategy to overcome drug resistance and improve treatment outcomes in ovarian cancer (Table 3). Conventional chemotherapy agents, such as platinum and taxane drugs, rely heavily on the induction of apoptosis [85]. In ovarian cancer cells, treatment stress can often induce cytoprotective autophagy, which can inhibit apoptosis and promote cell survival [62]. Bioactive natural compounds offer a unique advantage by simultaneously regulating autophagy and apoptosis through multi-targeted pathways, thus disrupting cancer cell adaptive response.

Bioactive natural substances demonstrate significant multi-bioactivities by concurrently targeting many signaling pathways that govern the autophagy-apoptosis axis in ovarian cancer. Curcumin and resveratrol are well-researched multi-target drugs that efficiently inhibit the PI3K/AKT/mTOR pathway, a key regulator of cancer cell survival, proliferation, and cytoprotective autophagy [86]. The inhibition of this pathway by these chemicals results in the reduction in mTOR-mediated autophagy suppression and the reinstatement of mitochondrial apoptosis via the activation of caspases and the modification of Bcl-2 family proteins [87]. Berberine and epigallocatechin gallate (EGCG) are significant activators of AMPK signaling, a metabolic sensor that inhibits mTOR and facilitates stress-induced autophagy while enhancing the susceptibility of cancer cells to apoptotic death [88,89]. The activation of AMPK by these drugs disturbs energy balance, promotes mitochondrial dysfunction, and amplifies apoptotic signaling in ovarian cancer cells [90]. Flavonoids like quercetin, luteolin, and apigenin exhibit multifaceted bioactivity by modulating the MAPK, p53, and NF-κB pathways, consequently inhibiting pro-survival signaling and reinstating apoptosis while optimizing autophagic flux [91]. These bioactive chemicals collectively influence the PI3K/AKT/mTOR, AMPK, and stress-response pathways, thereby disrupting survival mechanisms and underscoring their therapeutic promise as modulators of the autophagy-apoptosis axis in ovarian cancer.

Preclinical studies suggest that natural products can suppress pro-survival autophagy while restoring apoptotic potential, resulting in enhanced chemosensitivity. Curcumin and resveratrol can inhibit the PI3K/AKT/mTOR signaling pathway, leading to the modulation of autophagy and the induction of mitochondrial apoptosis [92]. Berberine and quercetin can activate AMPK and inhibit NF-κB signaling, promoting apoptotic cell death while inhibiting stress-induced autophagy [93]. These dual actions have been found to be particularly relevant in preclinical models of platinum-resistant ovarian cancer, where autophagy-mediated survival is a major resistance mechanism.

Clinically, the relatively low toxicity and dietary availability of many bioactive natural compounds make them attractive candidates for combination therapy. Several agents, including curcumin, resveratrol, and EGCG have already reached early-phase clinical trials in various cancers, showing an acceptable safety profile [94]. Nanotechnology-based delivery systems are also being developed to improve bioavailability and tumor targeting, thus overcoming one of the major limitations of natural products [95]. Although clinical evidence in ovarian cancer is currently limited, an increasing body of preclinical data strongly supports the inclusion of natural compounds that can modulate autophagy and apoptosis as adjuvants to standard chemotherapy [78]. Future clinical trials using biomarker-driven patient stratification and combination therapies will be essential to translate these promising results into effective treatments for ovarian cancer.

**Table 3:** Therapeutic and clinical relevance of bioactive natural products targeting autophagy-apoptosis crosstalk in ovarian cancer.

Bioactive Natural Product	Primary Molecular Targets	Autophagy Effect	Apoptosis Effect	Therapeutic/Clinical Implication	Ref.
**Curcumin**	PI3K/AKT/mTOR, AMPK	Suppresses cytoprotective autophagy	Activates mitochondrial apoptosis	Enhances platinum sensitivity, clinical safety established	[96]
**Resveratrol**	AMPK, p53, mTOR	Modulates stress-induced autophagy	Induces intrinsic apoptosis	Potential adjuvant therapy	[97]
**Berberine**	AMPK, mitochondrial pathways	Induces autophagic cell death	Activates intrinsic and extrinsic apoptosis	Effective in resistant models	[98]
**Quercetin**	NF-κB, AKT	Inhibits survival autophagy	Restores caspase activation	Overcomes apoptosis resistance	[99]
**EGCG**	PI3K/AKT, lysosomal pathways	Disrupts autophagic flux	Promotes mitochondrial apoptosis	Chemosensitization strategy	[100]
**Genistein**	AKT, estrogen receptor	Induces autophagy	Activates apoptosis	Targets hormone-linked signaling	[101]
**Sulforaphane**	HDACs, redox signaling	Context-dependent modulation	Induces apoptosis	Reverses cisplatin resistance	[81]
**Baicalein**	ERK, Beclin-1	Induces autophagy	Triggers intrinsic apoptosis	Active in platinum resistance	[56]
**Isoliquiritigenin**	ROS-mediated stress	Activates autophagy	Promotes apoptosis	Dual pathway targeting	[55]
**Betulinic acid**	Mitochondrial membrane	Autophagy-independent	Strong intrinsic apoptosis	Mitochondria-targeted therapy	[102]

## 5. Current Challenges, Limitations, and Future Perspectives

Despite the compelling preclinical data supporting the potential of bioactive natural products that modulate the autophagy-apoptosis crosstalk for the treatment of ovarian cancer, several critical challenges need to be addressed for their successful translation to the clinic. Insufficient bioavailability represents one of the main obstacles. Many natural compounds, including curcumin, resveratrol, quercetin, and epigallocatechin gallate, have poor water solubility, rapid metabolism, and limited absorption in the gastrointestinal tract [103]. These pharmacokinetic limitations result in low plasma and tumor drug concentrations that are far below the therapeutic levels, leading to a loss of efficacy in vivo despite promising in vitro data. In addition, extensive first-pass metabolism and rapid systemic clearance further limit the sustained drug exposure at the tumor site.

Another critical limitation relates to pharmacokinetics and target specificity. Natural products often affect multiple signaling pathways, which may be advantageous for modulating complex processes like autophagy and apoptosis but may also lead to unexpected off-target effects. The dual and context-dependent role of autophagy poses another challenge for its therapeutic manipulation. In ovarian cancer, autophagy can act as a cytoprotective mechanism that promotes cell survival under stress conditions, or as a form of programmed cell death when excessively activated [104]. Discriminating between cytoprotective and cytotoxic autophagy in different stages of the disease, genetic backgrounds, and therapeutic settings is challenging. Inadequate assessment of autophagic flux in preclinical studies may also lead to the misinterpretation of treatment outcomes.

Clinical translation is also hampered by tumor heterogeneity and the lack of validated prognostic biomarkers. Ovarian cancer exhibits significant inter- and intratumoral heterogeneity, and distinct molecular subtypes may respond differently to the modulation of autophagy and apoptosis [105]. The absence of reliable biomarkers to identify patients who are most likely to benefit from natural product therapies limits the design of effective clinical trials. Additionally, most of the available data are based on two-dimensional cell culture models, which fail to recapitulate the complexity of the tumor microenvironment, including hypoxia, metabolic stress, immune interactions, and extracellular matrix components that can significantly impact the autophagy-apoptosis crosstalk.

Nanotechnology-based drug delivery strategies offer potential solutions to many of these limitations. Nanocarriers such as liposomes, polymeric nanoparticles, solid lipid nanoparticles, and dendrimers can improve solubility, protect drugs from metabolic degradation, prolong circulation time, and enhance tumor accumulation through the enhanced permeability and retention effect [106]. Targeted nanodelivery systems based on ligands for receptors overexpressed in ovarian cancer may further improve specificity and therapeutic efficacy while minimizing systemic toxicity [107]. Co-delivery systems can also enable the simultaneous delivery of natural products with chemotherapeutic agents or autophagy inhibitors, thus enabling synergistic control of the autophagy-apoptosis crosstalk.

Combination regimens represent another particularly attractive future strategy. Natural products could be used as adjuncts to conventional chemotherapy, targeted therapy, or immunotherapy to block cytoprotective autophagy and sensitize cells to apoptosis. Rational combination approaches guided by molecular profiling may help overcome drug resistance and reduce treatment-related toxicity [108]. However, optimal dosing schedules, treatment sequences, and potential drug–drug interactions need to be carefully evaluated.

Future preclinical studies using three-dimensional spheroids, patient-derived organoids, and in vivo models are needed to validate the efficacy of these treatments in more therapeutically relevant settings. Early-phase clinical trials incorporating pharmacokinetic studies, biomarker-guided patient selection, and thorough assessment of autophagic flux and apoptotic markers are also equally important [109]. The integration of systems biology and precision medicine approaches will further refine therapeutic strategies. Overall, addressing these challenges will be critical for translating bioactive natural products that modulate the autophagy–apoptosis crosstalk into effective and clinically viable therapies for ovarian cancer.

## 6. Conclusion

Exploiting the crosstalk between autophagy and apoptosis is a viable strategy to overcome chemoresistance and improve therapeutic outcomes in ovarian cancer. Emerging evidence suggests that bioactive natural compounds can simultaneously modulate key signaling pathways that govern autophagy and apoptosis, thereby disrupting tumor cell survival mechanisms and restoring sensitivity to conventional treatments. Natural compounds such as curcumin, resveratrol, berberine, quercetin, and epigallocatechin gallate exhibit multitargeted activities and have favorable safety profiles, making them attractive candidates for adjuvant therapy approaches. Despite the promising preclinical findings, challenges related to bioavailability, pharmacokinetics, tumor heterogeneity, and translation to clinical settings remain. Advances in nanotechnology-based delivery systems, rational combination strategies, and biomarker-driven patient stratification are expected to improve therapeutic efficacy and clinical translation. The continued integration of mechanistic studies with advanced preclinical models and well-designed clinical trials will be essential to fully harness the potential of bioactive natural compounds as modulators of autophagy–apoptosis crosstalk in ovarian cancer.

## Data Availability

No new data were created or analyzed in this study.
